# World-Renowned “Swiss” Pediatricians, Their Syndromes, and Matching Imaging Findings: A Historical Perspective

**DOI:** 10.3390/children10101668

**Published:** 2023-10-09

**Authors:** Laura M. Huisman, Thierry A. G. M. Huisman

**Affiliations:** Edward B. Singleton Department of Radiology, Texas Children’s Hospital, Baylor College of Medicine, Houston, TX 77030, USA; huisman@texaschildrens.org

**Keywords:** Zellweger, Kartagener, Prader-Willi, Schinzel-Giedion, Fanconi, Joubert-Boltshauser, Poretti-Boltshauser, Langer-Giedion, syndromes, imaging, history

## Abstract

The goal of this manuscript is to present and summarize several rare pediatric syndromes (Zellweger syndrome, Kartagener syndrome, Prader-Willi syndrome, Schinzel-Giedion syndrome, Fanconi anemia, Joubert-Boltshauser syndrome, Poretti-Boltshauser syndrome, and Langer-Giedion syndrome) who have been named after luminary “Swiss” physicians (pediatricians, pediatric neurologists, or pediatric radiologists) who recognized, studied, and published these syndromes. In this manuscript, a brief historical summary of the physicians is combined with the key clinical symptoms at presentation and the typical imaging findings. This manuscript is not aiming to give a complete comprehensive summary of the syndromes, nor does it ignore the valuable contributions of many “Swiss” scientists who are not included here, but focuses on several rare syndromes that benefit from imaging data.

## 1. Introduction

The early and correct recognition of rare pediatric diseases is essential for decisions related to treatment options, the monitoring of treatment results, and the prevention of complications, as well as the estimation of prognosis and the counselling of patients, parents, and caregivers, including a risk estimation for recurrence in future pregnancies. Over the past century, multiple rare neonatal and pediatric syndromes have been recognized and identified based upon the clinical excellence of various giants in medicine. On many occasions, these syndromes have subsequently been named after the physicians who first described the clinical presentation. In addition, characteristic imaging findings have progressively been recognized on conventional radiography (CXR), ultrasound (US), computer tomography (CT), or magnetic resonance imaging (MRI). In addition, advanced imaging approaches allowed us to identify clinically silent abnormalities early during a patient’s life, including the correct linkage of the clinical symptoms, imaging findings, and underlying pathology and etiology. This manuscript aims to alert pediatric physicians about the importance of combining the clinical presentation, laboratory data, and genetic analysis with key imaging findings. Familiarity with the syndrome will also guide which imaging should preferably be used and which organs and systems must be examined in order to recognize the full spectrum of abnormalities. Finally, presenting a select group of rare pediatric syndromes whose primary commonality is that they have been described/recognized by “Swiss” physicians also emphasizes that the recognition of a syndrome relies on exceptional clinicians and a stimulating environment of collaborative academicians who share a long-term ongoing culture of discovery. In this manuscript, we exemplify this multidisciplinary approach by summarizing key clinical and imaging findings of Zellweger syndrome, Kartagener syndrome, Prader-Willi syndrome, Schinzel-Giedion syndrome, Fanconi anemia, Joubert-Boltshauser syndrome, Poretti-Boltshauser syndrome, and Langer-Giedion syndrome. We also briefly summarize how the name-givers of these syndromes all have shared roots at the University of Zurich, Switzerland, exemplifying the importance of a long-term tradition of discovery, collaboration, and clinical excellence.

## 2. Zellweger Syndrome

Hans-Ulrich Zellweger (1909–1990) was born in Lugano, Switzerland but spent his childhood growing up near the city of Chur. After completing medical school, he obtained his doctorate in Zurich in 1934 and went on to work under the great Albert Schweitzer in Lambarene, Gabon; and Guido Fanconi, who served as the physician-in-chief at the University Children’s Hospital Zurich. During his long career, Zellweger worked abroad at top universities in both the United States and Lebanon. Among these universities was the University of Iowa in Iowa City where he worked as a Professor of Pediatrics until his retirement in 1977. During his time at the university, he founded one of the first laboratories for clinical cytogenetics in the USA. He was passionate about research in the fields of neuromuscular disorders, genetic disorders, and various other dysmorphic syndromes and birth defects [1].

Zellweger syndrome (ZS), also known as cerebro-hepato-renal syndrome, is a rare recessive disease resulting from mutations in the peroxisome biogenesis factor (PEX) genes. Peroxisomes contain more than 50 enzymes that are involved with the catabolism and oxidation of very-long-chain and branched fatty acids that serve as a source of cellular energy. In addition, these enzymes are also involved in the synthesis of phospholipids and bile acids. The functional failure of the peroxisomes typically results in markedly elevated levels of very-long-chain fatty acids in the blood plasma. ZS is both the most severe and most common of peroxisomal disorders, presenting 1:50,000–100,000 in live births. Patients with ZS typically do not live longer than one year. Characteristic physical features of the syndrome include an abnormal face, characterized by a depressed hypoplastic nasal bridge, a towering and broad forehead, a large fontanelle, and deformed ears [2]. In addition to craniofacial abnormalities, eye abnormalities (large cornea), hepatomegaly, cryptorchidism, bilateral knee contractures, and chondrodysplasia punctata (stippled chondral calcifications) are also common. Furthermore, brain malformations, in particular, neuronal migration defects, are common. The peroxisomes are essential for normal brain formation, organization, and maturation. Newborn children affected by ZS typically present with hypotonia, seizures, the inability to feed, poor respiratory effort at delivery, and a lack of deep tendon reflexes [3].

**Figure 1:**
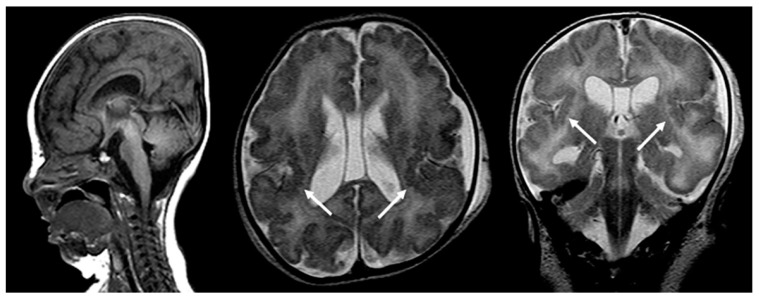
Sagittal T1-weighted, and axial and coronal T2-weighted MRIs of a neonate with confirmed Zellweger syndrome. Bilateral perisylvian polymicrogyria is noted (arrows), along with mild T2-hyperintense hypomyelination of the hemispheric white matter, mild ventriculomegaly, and frontal bossing.

Magnetic resonance imaging (MRI) of the brain typically shows a deficient, abnormal white matter myelination and various degrees of neuronal migrational and cortical organizational abnormalities consisting of regions of polymicrogyria, as well as areas of pachygyria (Figure 1). Other characteristic features include diminished or abnormal white matter myelination and a dysplastic cortical ribbon involving various parts of the cerebrum, in particular, the Sylvian and perisylvian region with a possible additional extension into the adjacent frontal and parietal lobes. It should be noted that the nature of the cortical abnormality, as well as the extent of the abnormality, differs among patients. Germinolytic cysts at the caudothalamic notch are another common finding among ZS patients. In addition, a laminar discontinuity of the principal olivary nucleus, as well as cerebellar heterotopias have been described [4,5].

**Figure 2:**
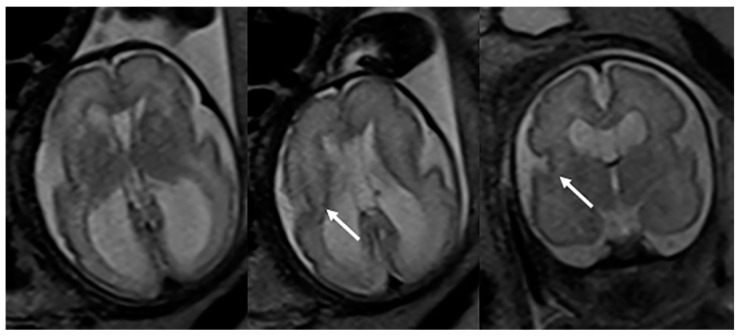
Axial and coronal T2-weighted fetal MRIs during the early third trimester of pregnancy show mild bilateral perisylvian polymicrogyria (arrows) and ventriculomegaly compatible with Zellweger syndrome (confirmed after delivery).

**Figure 3:**
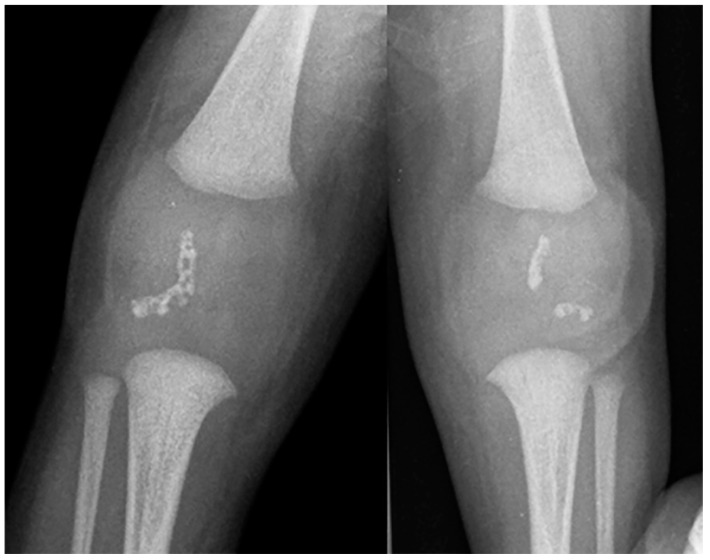
Frontal radiography of both knees in a 7-month-old male shows the characteristic crescent-shaped stippled chondral calcifications compatible with Zellweger syndrome.

Fetal MRI may allow for an early, prenatal diagnosis of ZS (Figure 2). Fetal MRI findings are similar to postnatal MRI which may reveal abnormal cortical gyration, delayed myelination or dysmyelination, and pseudocysts within the periventricular white matter. Furthermore, additional systemic imaging findings outside of the central nervous system may include renal microcysts and hepatosplenomegaly [6]. Additional radiographic findings (Figure 3) include stippled epiphyseal/chondral calcifications of the maturing skeleton, especially within the region of the knee (patella) and hip joints (triradiate acetabular cartilage) [7]. Zellweger syndrome is highly likely if elevated plasma very-long-chain fatty acids are noted in combination with imaging findings of bilateral peri-Sylvian pachy/polymicrogyria and chondrodysplasia punctate.

## 3. Kartagener Syndrome

Manes Kartagener (1897–1975) was a Polish-born physician who immigrated to Switzerland at the age of 18. He began studying medicine at the University of Zurich shortly after, obtaining his medical degree in 1924. After completing medical school, Kartagener began his residency at the Institute of Pathologic Anatomy in Zurich while working at the University Children’s Hospital Zurich as well. He continued his education by entering a second residency at the Institute of Dermatology, working at the University Institute of Physiological Chemistry, and training in internal medicine under Dr. Wilhelm Loffler in Zurich. After completing his training, he established a private practice in 1938, where he began to devote much of his time to researching bronchiectasis, a highly debated topic within the medical community at the time. Kartagener argued in support of a congenital rather than acquired basis for the disease, as he frequently noted the condition in conjunction with abdominal situs inversus [8].

Kartagener syndrome (KS) is a rare autosomal recessive disorder belonging to the group of primary ciliary dyskinesias (PCDs). Deficient ciliar motility is the etiological basis of KS with the simultaneous occurrence of situs inversus, chronic sinusitis, and bronchiectasis. A hallmark additional consequence of KS in males is infertility because of diminished sperm motility whereas females experience diminished fertility due to defective ovum transport. The early diagnosis of KS is essential as it allows patients the opportunity to consider treatment options for infertility. In addition to fertility issues, patients frequently experience recurrent chest infections, as well as ear, nose, and throat problems [9].

**Figure 4:**
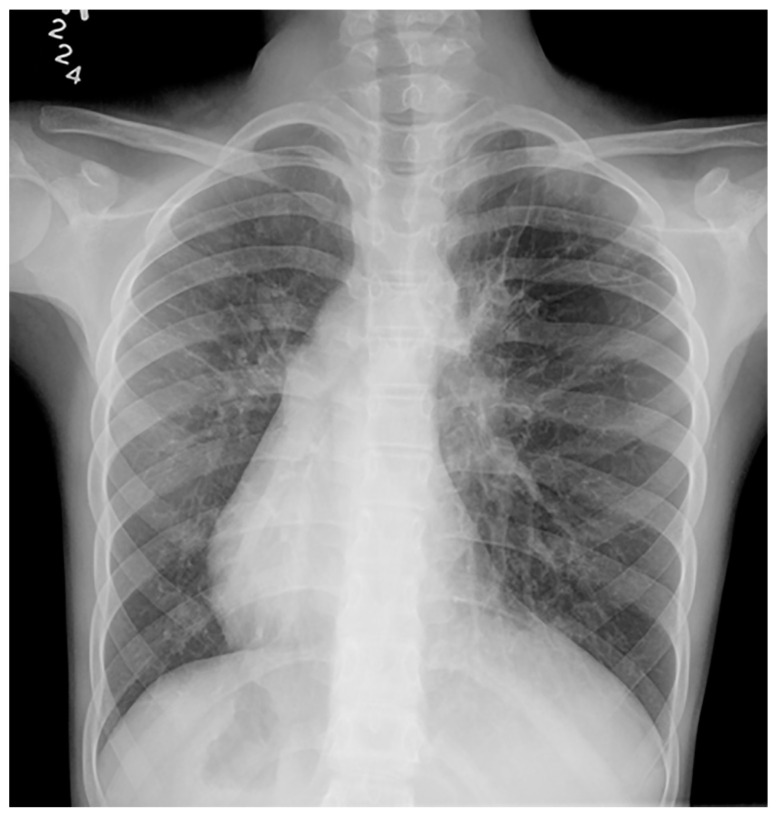
Frontal chest radiography in a young patient with confirmed Kartagener syndrome shows the characteristic complete situs inversus with dextrocardia and stomach on the right, and liver on the left within the abdomen. Bronchiectasis is noted in the left lung.

**Figure 5:**
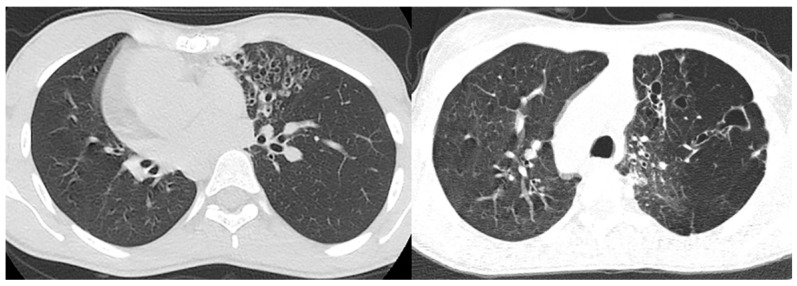
High-resolution CT of the lungs in two patients with confirmed Kartagener syndrome reveal the characteristic bronchiectasis (signet ring appearance) predominantly in the left lung.

**Figure 6:**
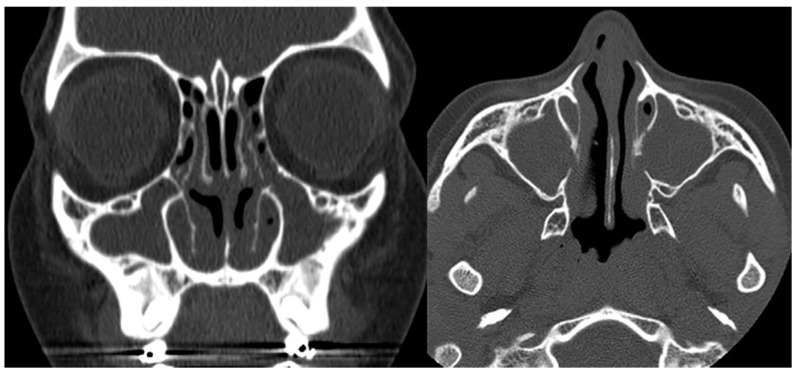
Coronal and axial CTs of the paranasal sinuses (bone window) in a patient with confirmed Kartagener syndrome show a complete, chronic opacification of the maxillary sinuses and parts of the ethmoidal air cells.

Imaging plays a vital role in identifying the various anatomical components of KS. Chest radiography easily confirms dextrocardia as part of a situs inversus bronchiectasis complex (Figure 4). Bronchiectasis may be suspected on chest radiography but is typically better identified by high-resolution chest computed tomography (HRCT) (Figure 5). HRCT imaging findings of bronchiectasis include abnormally dilated bronchi that are bigger than the adjacent bronchial arteries, creating a typical signet ring appearance. In addition, the normal tapering of the bronchial diameter towards the periphery is often absent. The complete transposition of thoracic and abdominal organs as seen on imaging confirms situs inversus [10]. Finally, small paranasal sinuses with or without findings compatible with acute and/or chronic sinusitis are best seen on a maxillofacial computed tomography (CT) study (Figure 6). No intracranial anomalies have been reported to be linked to KS.

## 4. Prader-Willi Syndrome

Andrea Prader (1919–2001) was born in Samedan, in the canton of Graubünden, but spent most of his life in Zurich. After completing medical school and working at various other institutions, Prader joined the University Children’s Hospital Zurich and became a mentee of Guido Fanconi. He later became the Chair of the Department of Pediatrics in Zurich, during which time he showed a significant interest in the field of endocrinology. During his tenure, he focused on researching intersex conditions, metabolic defects, growth and development, and the role of genetics. By the end of his career, Prader was a member of nearly all European Pediatric Societies and had been credited with several discoveries such as Prader-Willi syndrome, lipid adrenal hyperplasia, hereditary fructose intolerance, and pseudo-vitamin-D deficiency [11,12].

Heinrich Willi (1900–1971) was born in Chur as the sixth of nine children. He moved to Zurich to attend medical school and complete his residency at the Institute of Pathological Anatomy and the Winterthur Hospital Department of Medicine. In 1928, he joined the University Children’s Hospital in Zurich and became a mentee of Guido Fanconi. Willi became the Assistant Medical Director in Zurich in 1920. In 1936, after receiving a doctorate degree, he became the Director of Neonatology. As a neonatologist, Willi was interested in neonatal pathology and developmental abnormalities in infants born to diabetic mothers. Additionally, he conducted dietary research and studied the hematological effects of ascariasis, as well as leukemias of childhood [13].

Prader-Willi syndrome (PWS) is a genetic disorder, occurring in approximately 1:15,000 births, secondary to a partial deletion of chromosome 15 (15q11.2–q13 region) passed down by the father. The deletion is responsible for many of the symptoms associated with PWS, though the exact mechanism by which this occurs is not yet understood. Dysfunction of the hypothalamus is believed to play a role in the clinical presentation of PWS. Early features of PWS include severe hypotonia, poor appetite, and feeding difficulties. Excessive eating and progressive morbid obesity may follow in early childhood. Individuals with PWS also frequently present with behavioral difficulties and neuropsychiatric problems (obsessive compulsive personality disorder), mental delay, sleep difficulties, osteoporosis, and scoliosis. Genital hypoplasia, incomplete pubertal development, hypogonadism, and infertility are often part of the syndrome. Additional endocrine manifestations include growth hormone deficiency, hypothalamic pituitary dysfunction, type 2 diabetes mellitus, central hypothyroidism, and central adrenal insufficiency. Motor and language milestones are often delayed [14,15]. Scoliosis is seen in 40–80% of patients, likely secondary to the generalized hypotonia. Seizures may occur in young children but are usually refractory to seizure medication.

**Figure 7:**
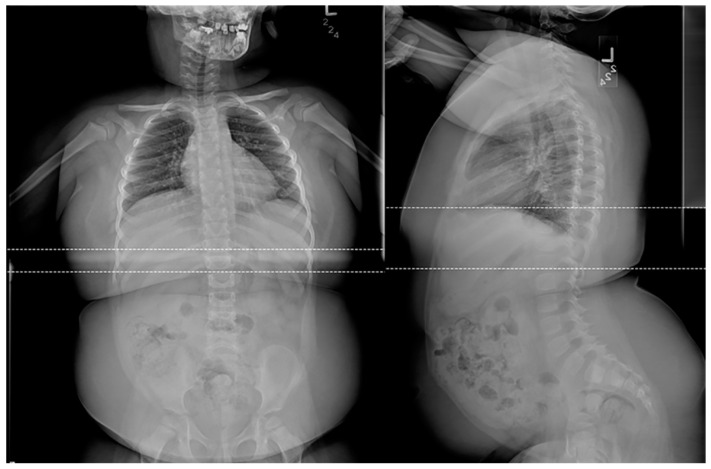
Frontal and lateral radiographs of a 4-year-old female patient with confirmed Prader-Willi syndrome show a significant adipositas, gracile, overtubulated humeri, and significant dental work secondary to advanced dental decay.

Calvarial abnormalities, a malformed sella turcica, advanced dental caries, small hands and feet with thin cortices and over-tubulated bones, hip dysplasia, and coxa valga are typically seen on radiography (Figure 7). The initial evaluation and follow-up monitoring of possible co-existing osteoporosis depend on serial dual-energy X-ray absorptiometry (DEXA) studies. Similarly, serial spine studies may be necessary in order to evaluate the progression of the scoliosis. Anatomical and functional MRIs have shown functional and structural brain alterations which are believed to be secondary to neuroendocrine and neurochemical alterations [15]. A recent study also identified smaller hypothalamic nuclei in patients with Prader-Willi syndrome. The significantly altered hypothalamic structure and resultant hypothalamic dysfunction are believed to be linked to the eating behavior [16].

## 5. Schinzel-Giedion Syndrome

Andres Giedion (1925–2013) was born to two famous art historians who, from an early age, influenced his understanding of shape and structure both in medicine as well as in design and architecture. Giedion enjoyed a classical humanistic education which included Latin and Greek. After finishing the Swiss gymnasium (high school) and graduating from the Zurich medical school, he went to Boston Children’s Hospital to undergo complete pediatric training under Charles Janeway. He later returned to Switzerland to work as part of the pediatric house staff at the University Children’s Hospital Zurich under the mentorship of Guido Fanconi. During this time, Giedion also completed radiology residency training under Hans Rudolf Schinz at the University Hospital of Zurich. Andres Giedion returned to Boston in 1958 for a fellowship in pediatric radiology under E.B.D. Neuhauser. After the completion of his fellowship, he returned in 1959 to Switzerland where he served as Chief of Radiology at the University Children’s Hospital Zurich. His doctorate thesis, “Cone shaped epiphyses: natural history and diagnostic impact in endochondral growth disturbance”, was approved by the medical faculty in Zurich in 1968 and he was promoted to the rank of professor in radiology in 1973. He served as Chief of Radiology at the Children’s Hospital in Zurich until his retirement in 1990 [17].

Albert Schinzel (1944–) is an Austrian born physician from Vienna. After completing medical school in Innsbruck, Vienna, and Berlin, he went on to work in both Finland and the United States before moving to Zurich. It was at the Institute of Medical Genetics at the University of Zurich where he became both a full professor and director in 1996. His strong interest in genetic research has led to five medical syndromes being associated with him and he continues to spread his medical knowledge with fellow professionals around the world [18].

Schinzel-Giedion syndrome (SGS) is a rare genetic disorder caused by heterozygous de novo mutations in the SETBP1 gene which is located on chromosome 18. The exact function of the SETBP1 gene is not yet fully understood. An etiological hallmark of this syndrome is the excessive accumulation of the SETBP1 protein which is believed to be secondary to a gain of function mutation. The increased concentration of the SETBP1 protein alters the gene expression regulation, which subsequently disrupts the normal development of multiple organs. The brain is especially affected by this mutation as the increased protein levels inhibit the proper development and function of neurons. As a result of this, a characteristic feature of SGS is severe neurodevelopmental delays and seizures. Additionally, children with SGS present with abnormal facial features (midface retraction, large forehead, hypoplastic nose, malformed ears, and widely set eyes), microcephaly, kidney, bladder issues, feeding problems, gastrointestinal problems, breathing problems, heart problems, impaired vision, alacrimia, hearing impairment, sleep disturbance, increased risk of cancer, skeletal abnormalities, and hypotonia. There are two forms of SGS: classical and atypical. Classical SGS patients have an average lifespan of 14–48 months, whereas atypical individuals live longer [19].

**Figure 8:**
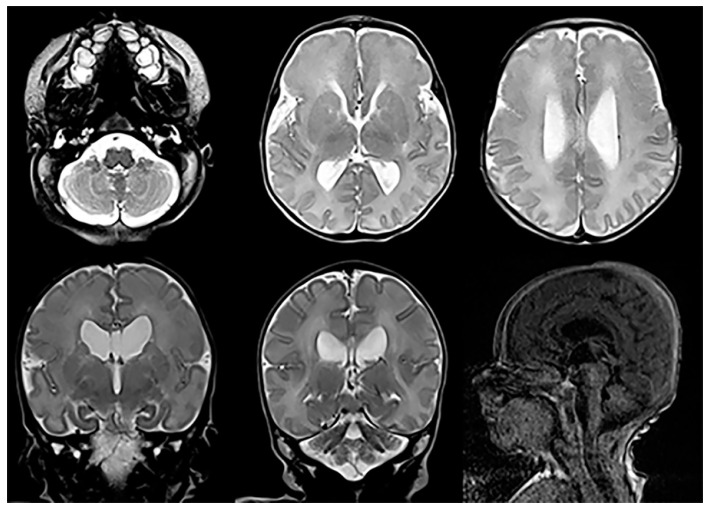
Axial and coronal T2-weighted and sagittal T1-weighted MRIs in a child with confirmed Schinzel-Giedion syndrome reveal mild cerebellar and vermian hypoplasia, a shallow pontine belly, and mild ventriculomegaly.

**Figure 9:**
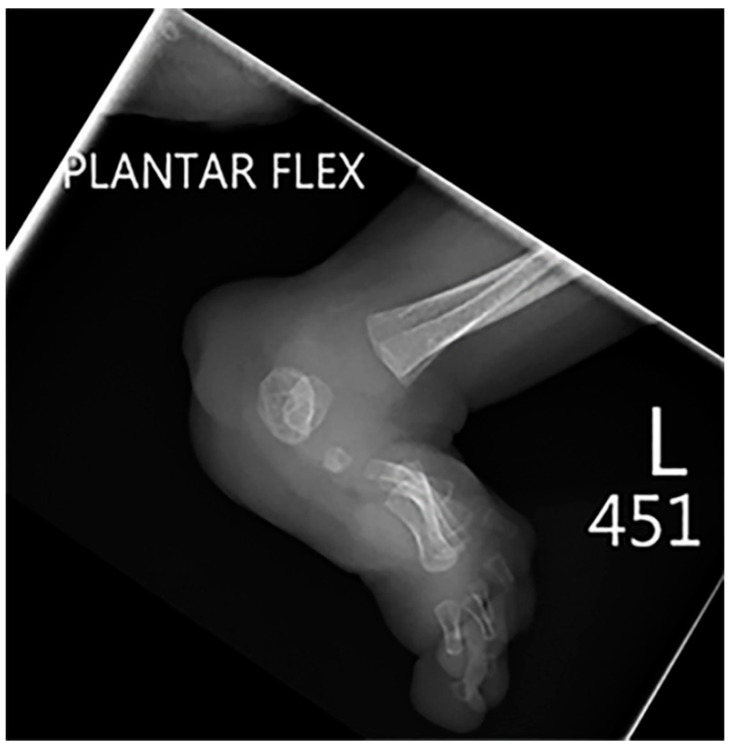
Lateral radiography of the same patient as shown in Figure 8 shows an extensive foot deformity.

Imaging of patients with SGS may reveal the following findings: hypertelorism, a steep, short skull base, inferior vermian hypoplasia, wide occipital synchondrosis, multiple wormian bones, hypoplastic first ribs, broadened ribs, mesomelic brachymelia, hypoplastic distal phalanges, hypoplastic/aplastic pubic bones, and congenital hydronephrosis (Figure 8). These findings may vary among patients [20]. SGS may also be suspected on a prenatal ultrasound or fetal MR based upon the correct recognition of characteristic features such as hydronephrosis, skeletal/limb abnormalities (Figure 9), nasal hypoplasia, prominent forehead, malformed ears, and ambiguous genitalia, and the final diagnosis may subsequently be confirmed by amniocentesis with molecular genetics [21].

## 6. Fanconi Anemia

Guido Fanconi (1892–1979) was born the youngest of six children in the Italian-speaking town of Poschiavo. After attending medical schools in Lausanne, Munich, Zurich, and Bern, Fanconi worked in the Departments of Pathology and Physiology at the University of Zurich. Upon completing his doctoral thesis, he completed a residency in Pediatrics at the Children’s Hospital of Zurich under Emil Feer. After serving as the senior pediatrician for some time, Fanconi was elected as the medical director of the hospital and appointed as a professor of pediatrics. His exceptional dedication to medicine is remembered by his many colleagues, peers, and numerous successful trainees. Guido Fanconi is remembered as one of the fathers of pediatrics in Switzerland [22].

Fanconi anemia is a rare, inherited disease secondary to a mutation in one of at least 15 different kinds of genes. The mutation causes the process of DNA repair to be slowed down. The accumulation of damaged DNA in the blood stem cells causes bone marrow failure; in particular, an impaired production of all three blood cell lines occurs. The disease is characterized by multiple physical abnormalities such as: café au lait spots, thumb and arm abnormalities, short stature, decreased birth weight, and microcephaly. Various organs/systems are also affected such as the central nervous system, the urogenital system, the gastrointestinal tract, and the heart. In addition to physical abnormalities, patients with Fanconi anemia are more likely to develop certain types of cancer. Leukemia or myelodysplasia are sometimes the first signs of the disease. Due to the lack of adequate numbers of erythrocytes, leukocytes, and platelets, patients may present with systemic symptoms such as extreme fatigue, frequent infections, easy ecchymosis, and epistaxis. Correct diagnoses are typically made within the first 10–15 years of life [23].

**Figure 10:**
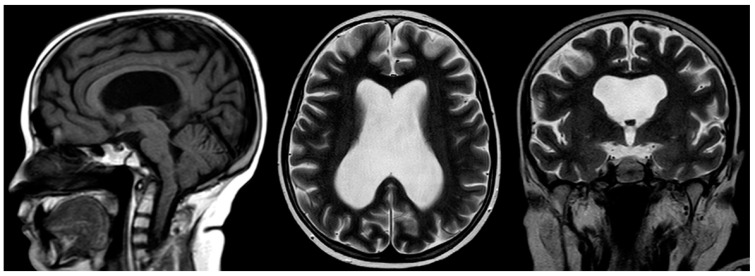
Sagittal T1-weighted, and axial and coronal T2-weighted MRIs of a 12-year-old male with confirmed Fanconi anemia. Mild basilary impression is noted with angulation at the cranio-cervical junction, small-sized pituitary gland, moderate supratentorial ventriculomegaly, and an absent septum pellucidum with box-like configuration of the lateral ventricles compatible with septo-optic dysplasia.

**Figure 11:**
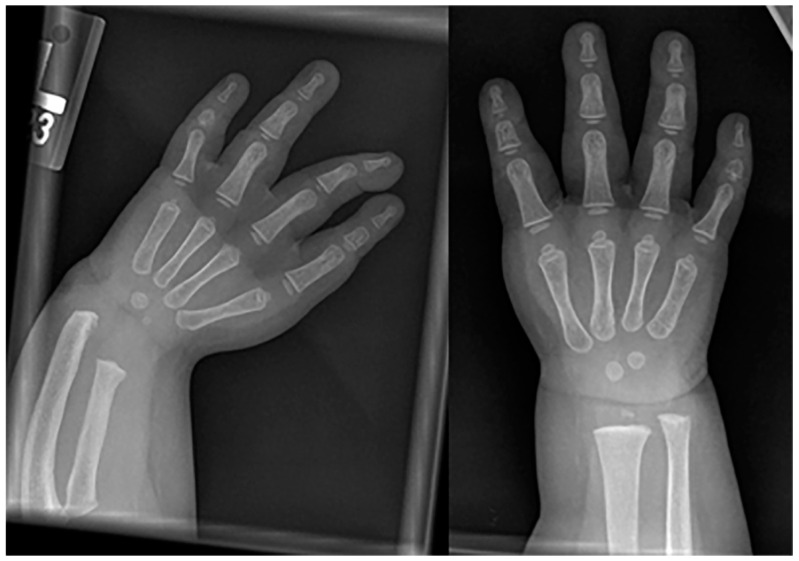
Frontal radiography of both forearms in a child with confirmed Fanconi anemia show left-dominant radial hypoplasia and bilateral aplasia of the thumbs.

**Figure 12:**
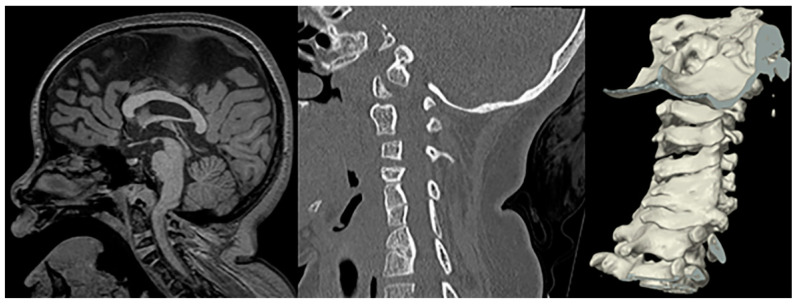
Sagittal T1-weighetd MRI and sagittal CT of the cervical spine including 3D surface rendering in a patient with confirmed Fanconi anemia show a narrowed foramen of magnum, mild impingement of the cranio-cervical junction, a short corpus callosum, and multilevel fusion anomalies of the cervical vertebral bodies.

Imaging of patients with Fanconi anemia is important in that it assists with endocrine assessment and allows for more informed decision making in clinical settings. Neuroimaging of patients with FA most often demonstrates congenital brain abnormalities in the form of abnormalities involving the pituitary gland, the posterior fossa, and the corpus callosum. The most common finding is a hypoplastic pituitary gland (Figure 10), [24]. In addition, neuroimaging may identify an ectopic neurohypophysis, platybasia, and other midline CNS abnormalities (Figure 10), [24]. Additional skeletal findings include radial ray anomalies (absent thumb), triphalangeal thumb, scoliosis, vertebral anomalies, and congenital hip dysplasia (Figure 11 and Figure 12). In addition, there is, based upon the deficient DNA repair mechanism, an increased risk of leukemia.

## 7. Joubert-Boltshauser Syndrome

Marie Joubert is a pediatric neurologist who first described Joubert syndrome while working at Montreal Children’s Hospital as a resident in the mid-1960s. Joubert had previously worked with Andre Barbeau, after which she decided to train in pediatric neurology at McGill University [25].

Eugen Boltshauser is an emeritus professor of pediatric neurology who served for many years as the Director of Pediatric Neurology at the University Children’s Hospital Zurich. He graduated from medical school in Zurich, and completed residencies in pediatrics, neurology, and pediatric neurology at the University Hospitals in Zurich including a two-year postgraduate training at The Hospital for Sick Children in London. Eugen Boltshauser is considered the name-giver of Joubert syndrome. After reading the landmark article published by Marie Joubert from McGill University describing a family with episodic hyperpnea, abnormal eye movements, ataxia, and developmental delay, which was presumed to be linked to an agenesis of the cerebellar vermis [26], Eugen Boltshauser published a paper on a second series of three children with the same unique clinical/neurological presentation, which he suggested should be named “Joubert syndrome” [27]. Over the many years of his career, Eugen Boltshauser remained highly involved in the research of cerebellar conditions, which eventually culminated in the textbook “Cerebellar disorders in children”, which he edited in collaboration with Jeremy Schmahmann [28].

**Figure 13:**
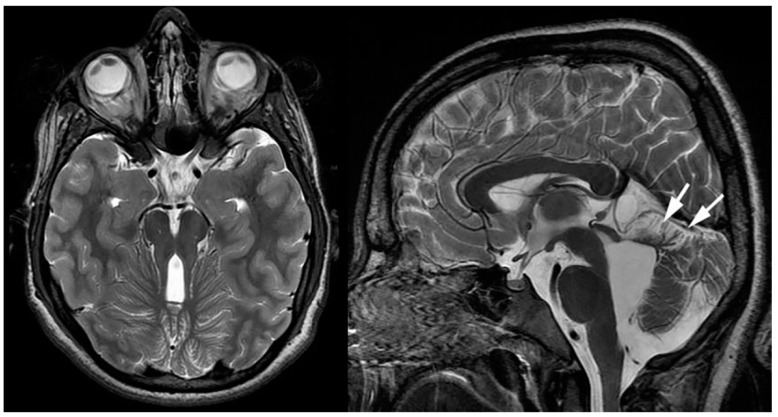
Axial and sagittal T2-weighted MRIs of a teenage patient with confirmed Joubert-Boltshauser syndrome show the characteristic molar tooth appearance of the brainstem on axial imaging with thickened and horizontal-coursing cerebellar peduncles and a nearly completely absent vermis. A small malformed/hypoplastic vermis is noted on the sagittal imaging (white arrows).

Joubert syndrome (JS) is a rare autosomal recessive disorder genetic disorder with the absence or insufficient growth of the cerebellar vermis, as well as an anomalous brain stem. A growing number of abnormal genes have been linked to Joubert syndrome. Joubert syndrome occurs when both parents carry a copy of one of the various causative gene mutations; it may, however, also occur spontaneously, that is, without any known family history. Currently, JS is recognized as a ciliopathy (deficient non-motile cilia) which may affect multiple organ systems within the body. Consequently, it is also known as cerebello-hepato-renal syndrome. Typical clinical symptoms include the following: hypotonia, abnormal eye movement, impaired intellectual development, ataxia, seizures, and hyperpnea, as well as kidney and liver abnormalities (nephronophtisis and liver fibrosis), and physical deformities such as polydactyly, cleft lip or palate, and tongue abnormalities. The term used to describe the characteristic posterior fossa brain abnormalities in JS is the “molar tooth sign” based upon the appearance of the brainstem on axial MR imaging resembling a molar tooth (Figure 13). The severity of the syndrome can range from mild, with limited motor challenges and normal development, to severe, with significant motor disabilities, moderately impaired mental development, and multiple organ impairments. There is no cure for JS, but affected children may benefit from therapies like infant stimulation, as well as physical, occupational, and speech therapy. To minimize the short- and long-term impact of JS, routine screening for progressing eye, liver, and kidney complications is advised [29].

The characteristic neuroimaging findings of JS are hypoplasia of the cerebellar vermis and the previously mentioned so-called “molar tooth sign” (MTS). The MTS as depicted on the axial cross-sectional imaging is a result of the abnormally horizontally running, thickened, and elongated superior cerebellar peduncles and an abnormally deep interpeduncular fossa (Figure 13). Additionally, a widening of the fourth ventricle and an enlargement of the posterior fossa may be seen. The typical abnormal posterior fossa morphology is seen in about 30% of patients. Additional supratentorial findings have been reported and include hippocampal malrotation, callosal dysgenesis, migrational anomalies, interpeduncular heterotopias, encephaloceles, and ventriculomegaly in various combinations and degrees of severity [29].

## 8. Poretti-Boltshauser Syndrome

Andrea Poretti (1977–2017) was born and raised in the city of Lugano, which is located in the canton of Ticino, an Italian-speaking part of Switzerland. He graduated from medical school in Bern, before moving to Zurich where he completed his medical thesis under the mentorship of Eugen Boltshauser and Michael Grotzer. His doctorate thesis focused on the complications, outcome, and quality of life of children with craniopharyngiomas. The research he conducted while writing his thesis inspired him to focus his future education and subsequent academic career on pediatric neurology. He returned to his hometown of Lugano for one year of pediatric residency, after which he moved to Zurich to complete his residency in pediatrics and training in pediatric neurology at the University Children’s Hospital. After completing his residency in 2010, Poretti moved to the USA to work at Johns Hopkins Hospital as a research fellow in the Division of Pediatric Neuroradiology under the mentorship of Thierry A. Huisman and remote mentorship of Eugen Boltshauser. During his time at Hopkins, he used his exceptional understanding of advanced, functional neuroimaging techniques to explore and study complex pediatric neurological disorders. He published more than 120 scientific papers during his time at Hopkins and was promoted to Assistant Professor of Radiology and Radiological Sciences and became the head of the Pediatric Neuroimaging Research mission within the Division of Pediatric Neuroradiology at Johns Hopkins. One year later, he was once again promoted to associate professor and joined the Kennedy Krieger Institute where he would have a dedicated clinic focusing on pediatric cerebellar disorders. Unfortunately, he passed away at the young age of 39 years. Poretti is remembered for his exceptional dedication to the classical tripartite academic mission of excellence in clinical care, teaching, and research and discovery [30].

**Figure 14:**
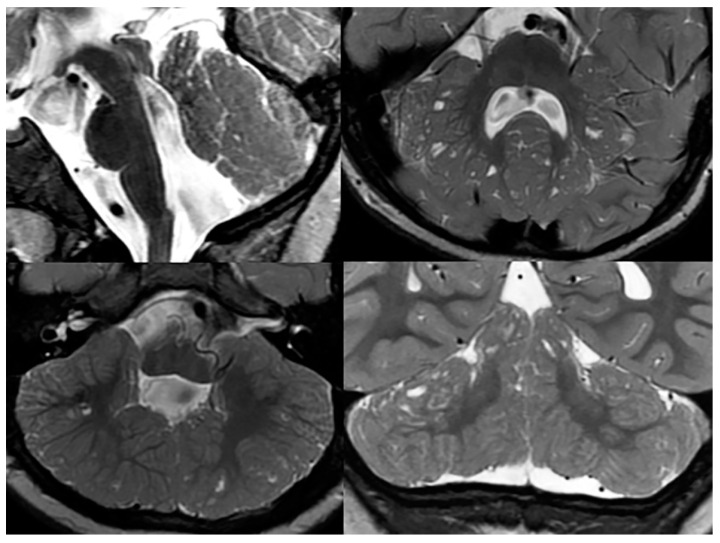
Sagittal, and axial and coronal T2-weighted MRIs of a patient with confirmed Poretti-Boltshauser syndrome show the characteristic elongation of the mesencephalon, disorganized cerebellar foliae, and multiple cerebellar inclusion cysts.

Poretti-Boltshauser syndrome (PBS) is a recessively inherited syndrome caused by mutations in the LAMA1 gene, which encodes for an extracellular matrix protein that is involved in basement membrane assembly essential for early brain development. The LAMA1 protein is, in particular, involved in the cell–cell and cell–extracellular-matrix adhesion, cell motility, and cell migration. A deficient LAMA1 protein typically results in a disruption of the cerebellar folia, especially [31]. Neuroimaging features of PBS resemble findings seen in α-dystroglyconopathies (cerebellar dysplasia with cysts); however, the clinical presentation in PBS is very different (Figure 14). Andrea Poretti recognized that, in PBS, typically, ataxia, intellectual disability, and ocular apraxia are noted, along with cerebellar cysts on neuroimaging [32]. In α-dystroglycanopathies, ataxia is usually absent, and ocular motor apraxia is rarely seen. Furthermore, in PBS, weakness is rare, serum creatine kinase is normal or only mildly increased, seizures are rare or absent, and no supratentorial malformations are noted, while in alpha-dystroglycanopathies, weakness is frequent, serum creatine kinase is markedly increased, seizures are frequent, and multiple supratentorial malformations occur. Furthermore, in PBS, impaired language skills and high degree of myopia or retinopathy may be noted. The research group of Dan Doherty identified bi-allelic mutations in the LAMA1 gene as the cause of this disease [33]. When these symptoms are present without any significant supratentorial anomalies and without muscular involvement, PBS is to be ruled out [34].

As mentioned before, the presence of cerebellar cysts is a characteristic finding in patients with PBS; however, the location, size, and number of cysts may vary. Cortical and subcortical cysts are most often found within the superior and anterior cerebellar vermis, and the superior and posterior cerebellar hemispheres. In addition to cysts, an abnormal, elongated, square-like fourth ventricle has been associated with PSB. Inferior cerebellar vermis hypoplasia and mild degrees of brainstem abnormalities are typically found in the majority of PBS children [34].

## 9. Langer-Giedion Syndrome

Leonard O. Langer (1928–2008) was an American radiologist born in Minnesota. After completing medical school in 1953, Langer pursued further radiology training at the University of Michigan. In 1966, he opened a private practice with suburban radiologic consultants. In addition to his private practice, Langer spent years working and researching as a professor at the Universities of Minnesota and Wisconsin. His work on bone deformities earned him worldwide recognition and he published widely in pediatric and genetic journals throughout his career. His work in co-operation with the Little People of America (LPA) led to a greater understanding of dwarfism in the medical community [35].

For information on Andres Giedion, please refer to the Schinzel-Giedion Syndrome section.

**Figure 15:**
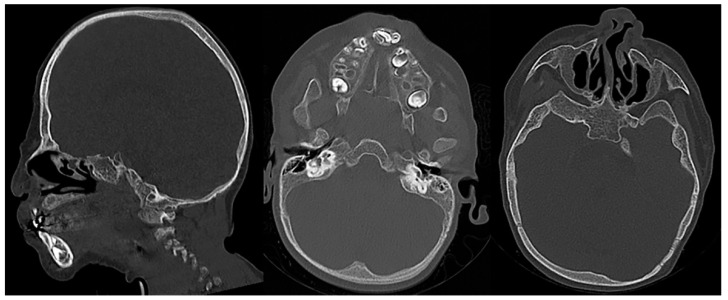
Sagittal and axial CT images (bone window) of a child with confirmed Langer-Giedion syndrome show the widening of the diploic space of the bony calvarium, extensive maxillofacial deformity with maxillary and mandibular hypoplasia, and a bulbous deformed nasal tip.

**Figure 16:**
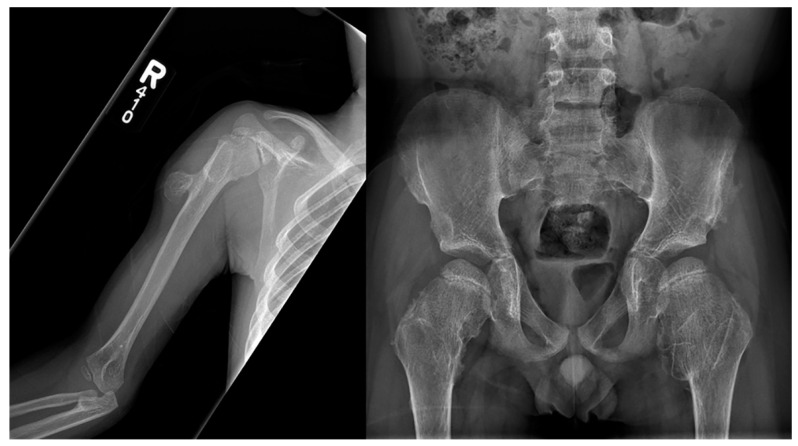
Frontal radiography of the right humerus and pelvis in a patient with confirmed Langer-Giedion syndrome show multiple exostoses along the proximal right humeral shaft, both femoral necks, and both iliac bones. The bones also appear demineralized.

**Figure 17:**
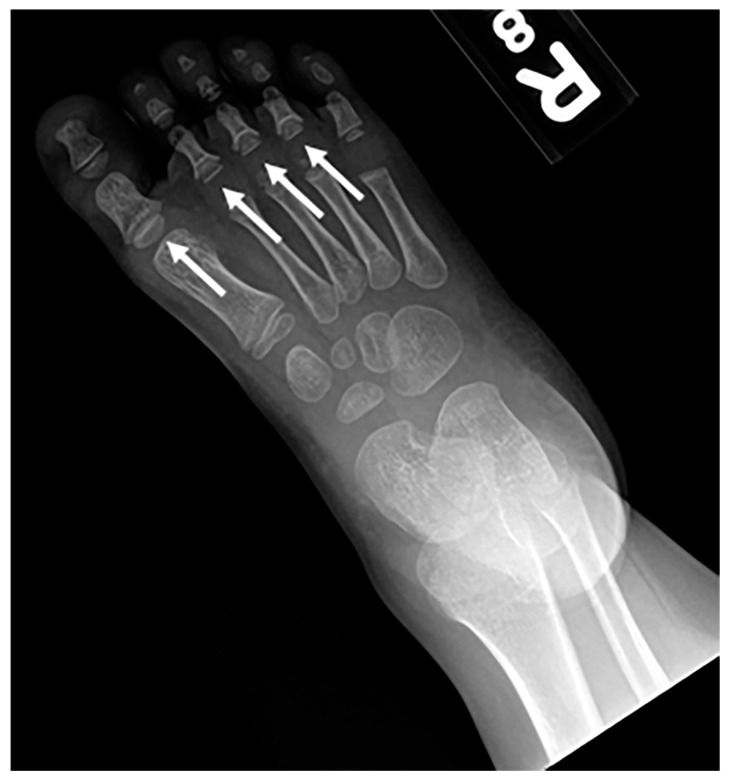
Frontal radiography of the right foot in a patient with confirmed Langer-Giedion syndrome show multiple cone-shaped epiphysis of the proximal phalanges (arrows).

Langer-Giedion syndrome (LGS), also known as trichorhinophalangeal syndrome type II (TRPS2), is a rare autosomal dominant genetic disorder characterized by skeletal abnormalities, facial malformation, and brittle hair. It is caused by a chromosome 8 contiguous deletion; this chromosome is involved in the normal development of multiple organs. Typical features of LGS include sparse/brittle hair, cone-shaped epiphyses of the extremities, numerous cartilaginous exostoses, bulbous nasal tip, large protruding ears with thickened ear cartilage, upturned nares, prominent philtrum, and mild mental delay (Figure 15, Figure 16 and Figure 17). Clinical presentation of LGS is highly variable and, as a result, the syndrome often remains undiagnosed. To accurately diagnose LGS, clinical examination, radiographic imaging, and genetic investigations should be performed. The proper identification of LGS is essential in order to ensure the establishment of appropriate supportive management, particularly in the prevention of orthopedic complications [36,37,38].

The presence of ivory epiphysis, type 12A coned epiphysis, and exostosis in radiographs indicates a potential connection to LGS, although additional research is needed to confirm this triad’s diagnostic significance. Since radiological features in the hands may evolve over time, obtaining follow-up hand radiographs is valuable in cases where LGS is suspected based on clinical signs, but the initial imaging results are negative or inconclusive [39].

## 10. Conclusions

The road to the discovery of rare diseases and their accurate and timely diagnosis relies on a culture of curiosity, professionalism, collaboration, clinical excellence, and teamwork. The early and correct recognition of rare pediatric, multi-system diseases requires a multi-disciplinary approach in which family history, clinical presentation, laboratory data, genetics, and imaging findings are integrated. It is, of course, also a sine qua non that physicians should be familiar with these rare diagnoses. In this manuscript, multiple rare, pediatric, multisystem diseases which may be suspected early on, sometimes even before the child becomes symptomatic, are being discussed against the background of the professional development of the name-givers who all worked at some time in their career at a university known for its culture of discovery and excellence. The presented cases are excellent examples of how a stimulating culture supports ongoing medical discovery.

## Data Availability

Not applicable as no patient interaction or patient identifiable data are presented, inlcuded or analyzed.
